# MPC1 promotes the damage of human coronary endothelial cells in macrolide-resistant mycoplasma pneumoniae via inhibiting mitophagy

**DOI:** 10.3389/fped.2026.1731155

**Published:** 2026-04-29

**Authors:** Yalin Fu, Minmin Li, Qiurong Chen, Rong Ding, Tian Hu, Qiao Wu, Shumei Peng

**Affiliations:** Department of Pediatrics, Guangdong Women and Children Hospital, Guangzhou, Guangdong, China

**Keywords:** macrolide-resistant *M. pneumoniae*, mitochondrial pyruvate carrier 1, mitophagy, pyroptosis, treatment

## Abstract

**Introduction:**

*Mycoplasma pneumoniae* (*M. pneumoniae*) is a common community-acquired pneumonias among children and young adults. Long-term use of macrolide antibiotics treatment contributes to Macrolide-resistant *M. pneumoniae* (MRMP). This study aimed to investigate the roles of mitochondrial pyruvate carrier 1 (MPC1) in MRMP.

**Methods:**

Human coronary endothelial cells (HCAECs) were co-cultured with MRMP. mRNA expression was calculated using quantitative reverse transcriptase PCR (qRT-PCR). Protein expression was detected using Western blot. Cytokine release was detected using enzyme-linked immunosorbent assay. The morphology of mitochondria was detected using transmission electron microscopy assay. The viability of HCAECs was determined using cell counting kit-8 assay. Cytotoxicity was determined using lactate dehydrogenase cytotoxicity assay. Cell death was analyzed using terminal deoxynucleotidyl transferase (TdT) dUTP Nick-End Labeling (TUNEL) assay.

**Results:**

We found that MRMP exposure mediated mitochondrial damage and pyroptosis of HCAECs. Moreover, MPC1 was overexpressed in HCAECs exposed to MRMP. Inhibition of MPC1 promoted mitophagy as well as suppressed the pyroptosis of HCAECs. However, blocking mitophagy signaling antagonized the effects of MPC1 deficiency, resulting in mitochondrial damage and pyroptosis of HCAECs.

**Conclusion:**

MPC1 promotes mitochondrial damage and pyroptosis of HCAECs in MRMP through inhibiting mitophagy. Therefore, targeting MPC1 may be a promising strategy for MRMP.

## Introduction

*Mycoplasma pneumoniae* (*M. pneumoniae*) is a cell-wall–deficient, genome-condensed bacterium (1). *M. pneumoniae* minimal metabolic repertoire forces intimate dependence on host nutrients, making macrolide antibiotics (2). Moreover, the progression of *M. pneumoniae* promotes the progression of systemic vasculitis, such as Kawasaki disease (3). The incidence of macrolide-resistant *M. pneumoniae* (MRMP) is increasing (4). Resistance is almost exclusively conferred by single-nucleotide transitions in domain V of the 23S rRNA (5, 6). The limited paediatric safety data for tetracyclines and fluoroquinolones leave clinicians with few alternatives (7, 8). Therefore, there is an urgent need for adjunctive or host-directed strategies for MRMP.

Mitochondria are integrators of innate immunity and metabolic stress (9). Upon bacterial infection, mitochondria release mitochondrial DNA (mtDNA) into the cytosol to activate cyclic GMP-AMP synthase/stimulator of interferon response cGAMP interactor signalling, mediating NLR family pyrin domain containing 3 (NLRP3) inflammasome assembly (10–12). Intracellular bacteria actively remodel host mitochondrial networks to escape these defenses (13, 14). Mycobacterium tuberculosis, Legionella pneumophila and Chlamydia trachomatis induce fragmentation of the mitochondrial reticulum and dampen OXPHOS, or usurp mitophagy (15–18). Interestingly, inhibition of mitochondrial ROS alleviates the progression of *M*. *pneumoniae* pneumonia (19). However, whether *M. pneumoniae* manipulates mitochondrial quality-control pathways is unknown.

Mitochondrial pyruvate carrier 1 (MPC1) gates cytosolic pyruvate into the mitochondrial matrix (20, 21). MPC1 dysfunction skews the NAD^+^/NADH ratio, increases lactate production and reprograms immune-metabolic responses (22). Emerging evidence indicates that MPC activity also dictates mitophagy flux (23, 24). Conversely, MPC1 over-expression contributes to mitochondrial dysfunction and ferroptosis in cervical cancer (25).

We recently observed that MPC1 promotes MRMP-induced mitochondrial dysfunction. This raises the hypothesis that MPC1 constitutes to a host metabolic checkpoint that confines intracellular bacteria. In the present study, we, therefore, sought to: (i) dissect the molecular circuitry linking MPC1 to mitophagy during MRMP infection; (ii) determine whether MPC1-dependent mitophagy modulates macrolide efficacy; and (iii) explore pharmacological targeting of the MPC1–mitophag*y* axis.

## Materials and methods

### Cell culture

Human coronary endothelial cells (HCAECs) were purchased from ATCC, USA. Cells were cultured in RPMI-1640 (12633020; Gibco, USA) containing 10% fetal bovine serum (FBS) (A5669701; Gibco, USA).

Bacteria were washed twice in phosphate-buffered saline (PBS) (FB04109050; Thermo Fisher Scientific, USA), resuspended in infection RPMI-1640 medium with 2% FBS, and added at a multiplicity of infection (MOI) of *M. pneumoniae* (DSM22911; HuizaoBio, China) at the content of 10(6) color-changing units (CCU) per cell. Plates were centrifuged (200×*g*, 5 min) to synchronize contact and incubated at 37 °C, 5% CO_2_ for the indicated times.

HCAECs were treated with an MPC1 inhibitor UK-5099 (20 μM) or proteasome inhibitor MG132 (2.5 μM).

### Air-liquid interface (ALI) infection

HCAECs were apically infected with 10^7^ CCU in 100 µL PBS. After 2 h adsorption, the inoculum was removed and basolateral medium refreshed. Transepithelial samples were harvested by PBS + 0.05% Triton X-100 lavage (ST797-100 mL; Beyotime, China).

### Infection protocol

Log-phase MRMP or M129-R were washed twice in PBS, resuspended in infection medium, and added at MOI = 10 CCU per cell (unless specified). To synchronize contact, plates were centrifuged (200×*g*, 5 min) and incubated at 37 °C, 5% CO_2_ for indicated times. Extracellular bacteria were removed at 4 h post-infection by two PBS washes ± 100 µg·mL^−1^ gentamicin (2 h) to quantify intracellular burden.

### Transmission electron microscopy (TEM)

Cells were fixed, dehydrated and embedded. 70-nm sections were stained with uranyl acetate and lead citrate, then imaged on a JEM-1400Plus (JEOL; Keyence, Japan) at 120 kV. Mitochondrial area, cristae integrity, and bacterial proximity were quantified in a blinded fashion (≥50 micrographs per group).

### Enzyme-linked immunosorbent assay (ELISA)

Interleukin 1β (IL-1β) and IL-18 levels were measured using DuoSet ELISA kits (DLB50 and NBP3-39618; R&D Systems, USA). Absorbance was determined using a SpectraMax i3x plate reader (293817527; Molecular Devices, USA) at the wavelength of 450 nm (correction 540 nm).

### Quantitative reverse transcriptase PCR (qRT-PCR)

Total RNA was extracted from HCAECs using TRIzol reagent (15596026CN; Invitrogen, USA). cDNA was synthesized using a PrimeScript™ II 1st Strand cDNA Synthesis Kit (6210A; Takara, Japan). PCR was performed using PrimeScript™ II High Fidelity RT-PCR Kit (R023A; Takara, Japan). Gene expression was quantified on a QuantStudio 7 Flex using SYBR Select (Thermo Fisher Scientific, USA) and the 2-*^ΔΔ^*^Ct^ method, normalizing to β-actin.

### Western blotting

HCAECs were lysed in radioimmune precipitation assay buffer (P0013B; Beyotime, China). Protein was collected, concentrated, and resolved on 15% gradient sodium dodecyl sulfate-polyacrylamide gel electrophoresis. After transferred to polyvinylidene fluoride, protein was probed with antibodies against MPC1 (16118-1-AP; Proteintech, USA), parkin RBR E3 ubiquitin protein ligase 2 (PARK2) (4,211; Cell Signaling Technology, USA), PTEN induced kinase 1 (PINK1) (NBP1-71769; Novus, USA), LC3B (2,775; Cell Signaling Technology, USA), anti-NLRP3 (ab263899; Abcam, UK), PYD and CARD domain containing (ASC) (67,824; Cell Signaling Technology, USA), cleaved caspase 1 (CL-CASP1) (ab238972; Abcam, UK), and β-actin (A2228; Sigma-Aldrich, Germany), and then with goat-anti-rabbit (ab6721; Abcam, UK) and goat-anti-mouse (ab6789; Abcam, UK) secondary antibodies. The bands were pictured using an enhanced chemiluminescence kit (ab133406; Abcam, UK). Densitometry was performed in ImageJ.

### Cell counting kit 8 (CCK-8) assay

After MRMP treatment, HCAECs (2 × 10^3^ per well, 96-well plate) were treated with 10 µL CCK-8 reagents (CK04-100T; Dojindo, Japan). Absorbance was measured at 450 nm. Viability was calculated relative to uninfected, vehicle-treated control (set to 100%). Each condition was run in octuplicate; three independent experiments were performed.

### Lactate dehydrogenase (LDH) cytotoxicity assay

After MRMP treatment, supernatants were harvested. LDH levels were determined using the CyQUANT™ LDH kit (C20301; Thermo Fisher Scientific, USA).

### Terminal deoxynucleotidyl transferase (TdT) dUTP nick-end labeling (TUNEL) staining

HCAECs were fixed and permeabilised. Cell death was determined using a Click-iT™ Plus TUNEL Assay (C10617; Thermo Fisher Scientific, USA). Nuclei were counterstained with 1 µg/mL 4′,6-diamidino-2-phenylindole (C1005; Beyotime, China). Images were pictured using a microscope (Zeiss, Germany).

### Statistical analyses

Statistical analysis was conducted on GraphPad Prism 9.5.1. Data were evaluated by analysis of variance followed by Tukey's post-test. *P* < 0.05 was considered statistically significant. All numerical data are presented as mean ± standard deviation from at least three biological replicates.

## Results

### MRMP mediates upregulation of MPC1

**Figure 1:**
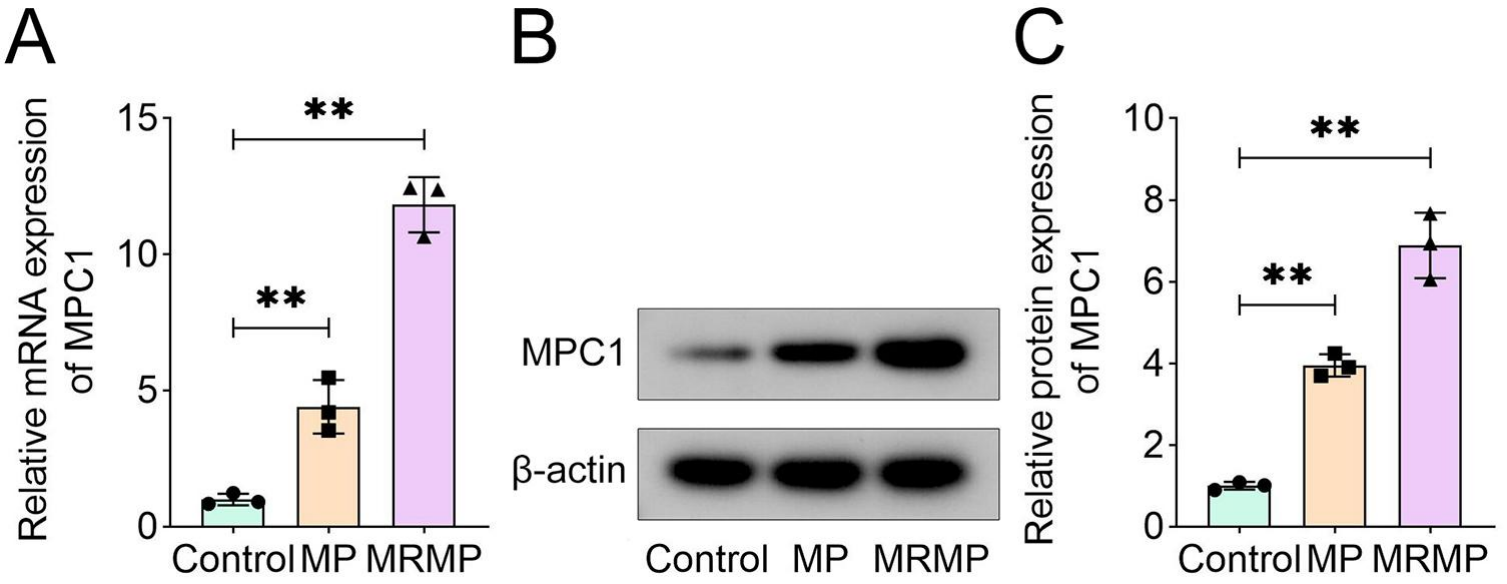
MRMP mediates upregulation of MPC1. **(A)** MPC1 mRNA expression in MRMP-infected HCAECs was determined using qRT-PCR (*n* = 3). **(B,C)** MPC1 protein expression in MRMP-infected HCAECs was determined using Western blot (*n* = 3). **p < 0.01, one-way ANOVA followed by Tukey's *post hoc* test.

MPC1 plays a pro-inflammatory role in fever and Alzheimer's disease (26, 27). To confirm the roles of MPC1 in MRMP, we determined its expression in *in vitro* MRMP models. As shown in Figure 1A, MRMP significantly increased MPC1 mRNA expression compared with *M. pneumoniae* group (Figure 1A). This is consistent with the results from Western blot. MRMP significantly increased MPC1 protein expression compared with *M. pneumoniae* group (Figures 1B,C).

### UK-5099 alleviates MRMP-induced mitochondrial damage

**Figure 2:**
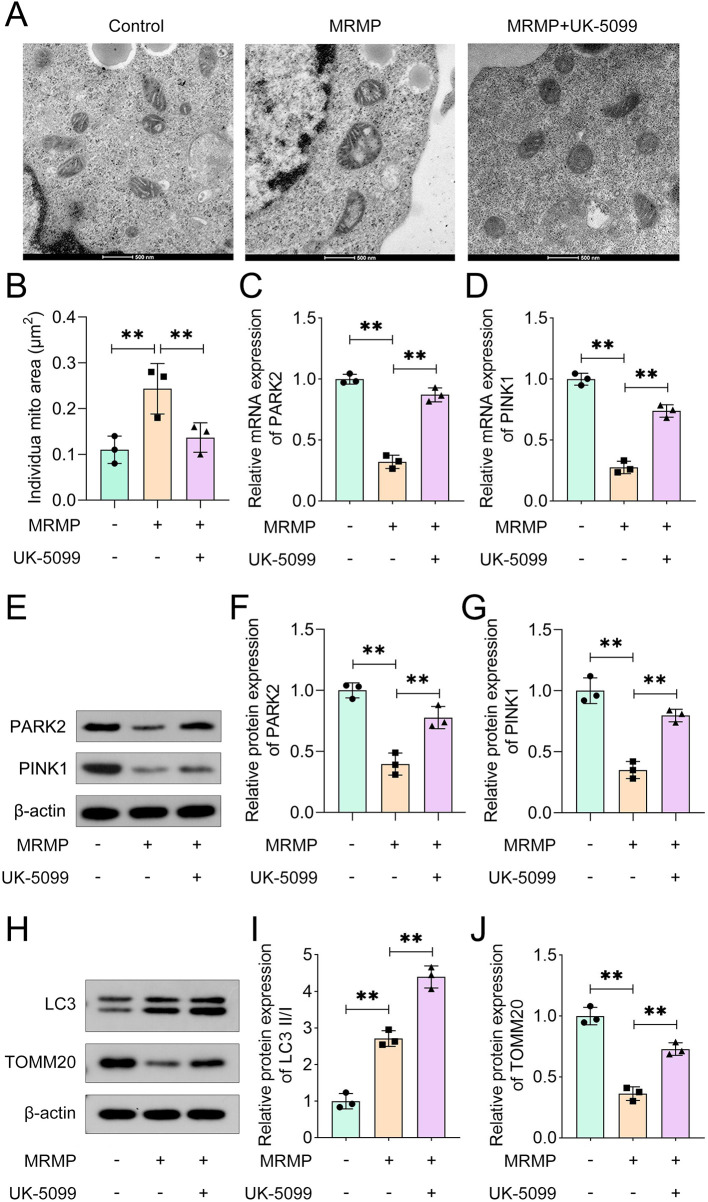
UK5099 alleviates MRMP-induced mitochondrial damage **(A)** mitochondrial morphology was imaged using TEM (*n* = 3). Scale bar: 500 nm. **(B)** Quantification of the average individual mitochondrial area (*n* = 3). **(C,D)** mRNA levels in MRMP-infected HCAECs were determined using qRT-PCR (*n* = 3). **(E–J)** Protein expression in MRMP-infected HCAECs was determined using Western blot (*n* = 3). **p < 0.01, one-way ANOVA followed by Tukey’s *post hoc* test.

To confirm the roles of MPC1 in MRMP, HCAECs were treated with UK-5099 (an inhibitor of MPC1). UK-5099 treatment markedly inhibited the swelling of mitochondria induced by MRMP (Figure 2A). MRMP significantly increased the individual mitochondrial area (Figure 2B), which was reversed by UK-5099. The mRNA expression of PARK2 and PINK1 in MRMP group was significantly reduced compared with control group (Figures 2C,D), which was markedly enhanced by UK-5099. Moreover, MRMP significantly inhibited the protein expression of PARK2 and PINK1 (Figures 2E–G), which was markedly reversed by UK-5099. MRMP-induced downregulation of TOMM20, suggesting the dysfunction of mitochondria (Figures 2H–J). These results suggested that inhibition of MPC1 improves mitochondrial function via mediating mitophagy.

### UK-5099 alleviates MRMP-induced pyroptosis of HCAECs

**Figure 3:**
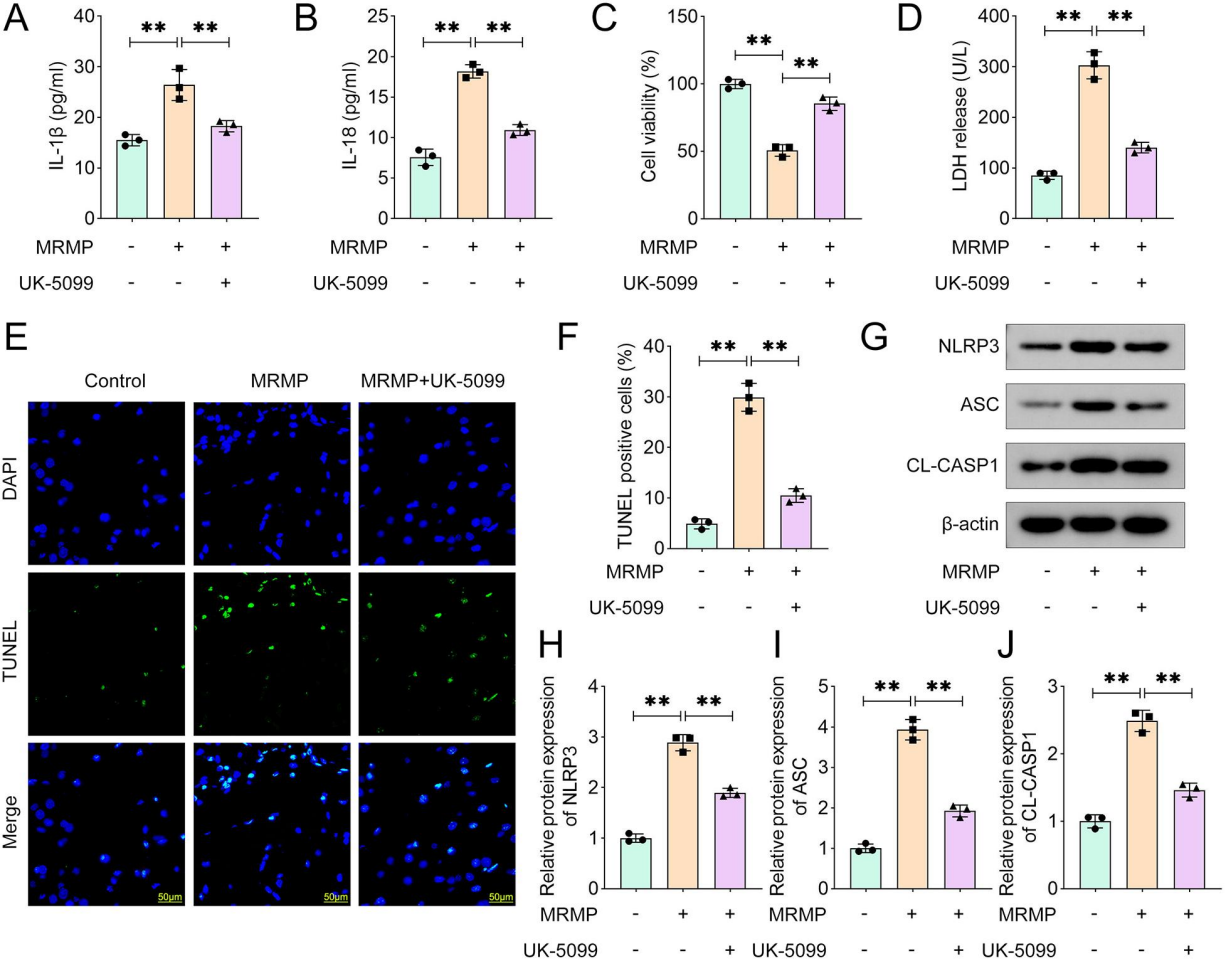
UK5099 alleviates MRMP-induced pyroptosis of HCAECs. **(A,B)** Cytokine release was determined using ELISA (*n* = 3). **(C)** Cell viability was determined using CCK-8 assay (*n* = 3). **(D)** Cytotoxicity was determined using LDH assay. **(E,F)** Cell death was determined using TUNEL assay (*n* = 3). Scale bar: 50 μm. **(G–J)** Protein expression in MRMP-infected HCAECs was determined using Western blot (*n* = 3). **p < 0.01, one-way ANOVA followed by Tukey's *post hoc* test.

MRMP-induced tissue damage is frequently accompanied with inflammation-related cell death, especially pyroptosis. We found that MRMP significantly increased the release of IL-1β and IL-18 (Figures 3A,B), which was markedly reversed by UK-5099. UK-5099 significantly alleviated the effects of MRMP and increased the cell viability of HCAECs (Figure 3C). Moreover, UK-5099 significantly inhibited MRMP-induced cytotoxicity of HCAECs (Figure 3D). UK-5099 significantly reduced the percentages of TUNEL positive HCAECs (Figures 3E,F). Additionally, UK-5099 significantly reversed the effects of MRMP and inhibited the activation of NLRP3 inflammasome (Figures 3G–J). These results suggested that MPC1 deficiency alleviates MRMP-induced pyroptosis of HCAECs.

### MG132-mediated PINK1 deficiency induces mitochondrial damage

**Figure 4:**
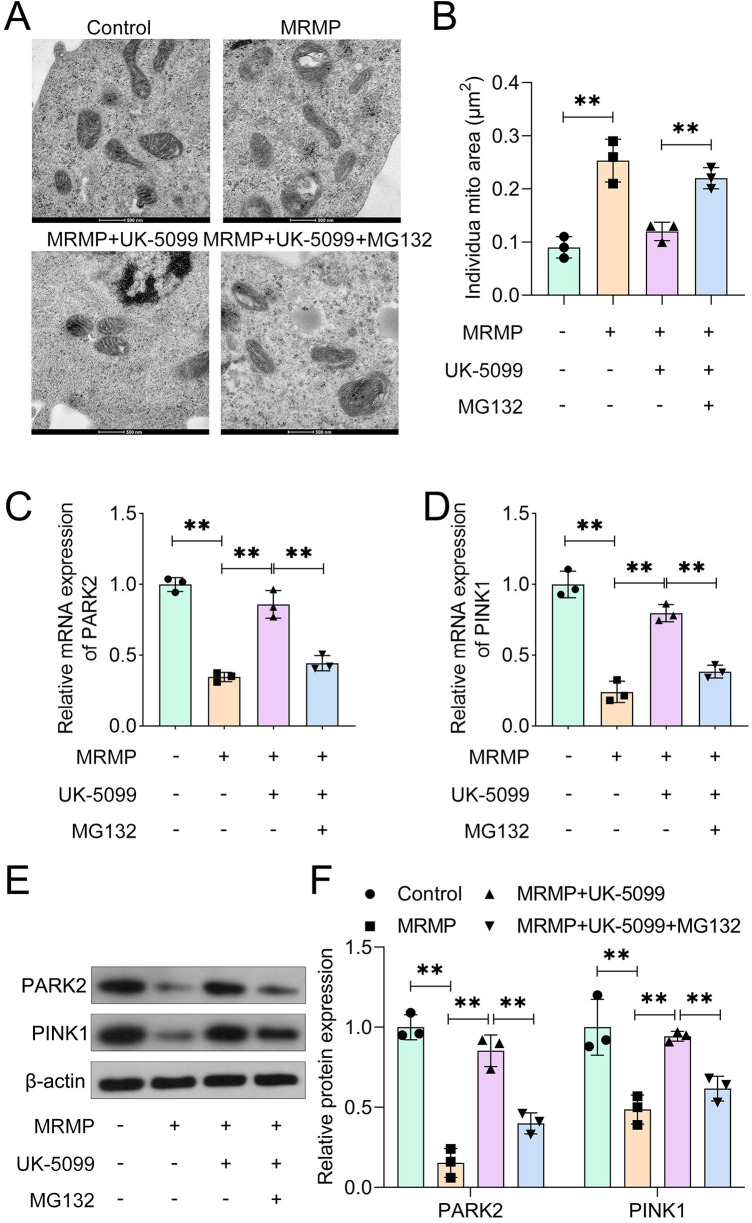
MG132-mediated PINK1 deficiency induces mitochondrial damage. **(A)** Mitochondrial morphology was imaged using TEM (*n* = 3). Scale bar: 500 nm. **(B)** Quantification of the average individual mitochondrial area (*n* = 3). **(C,D)** mRNA levels in MRMP-infected HCAECs were determined using qRT-PCR (*n* = 3). **(E,F)** Protein expression in MRMP-infected HCAECs was determined using Western blot (*n* = 3). **p < 0.01, one-way ANOVA followed by Tukey's *post hoc* test.

Mitophagy is a self-defense system that protects cells from stress response and damage (28). The activation of mitophagy signaling inhibits pyroptosis in pulmonary fibrosis, sepsis-induced acute lung injury as well as *Klebsiella pneumoniae* infection. To confirm this, HCAECs were treated with MG132 (an inhibitor of PINK1) in the background of UK-5099 culture. Compared with MRMP plus UK-5099 group, MG132 treatment significantly induced the damage of mitochondria (Figure 4A), as well as increased the individual mitochondrial area (Figure 4B). MG132 significantly reduced the mRNA expression of PARK2 and PINK1 (Figures 4C,D). These results suggested that inactivation of mitophagy signaling induces mitochondrial damage in HCAECs. Moreover, MG132 significantly inhibited the protein expression of PARK2 and PINK1 (Figures 4E,F).

### MG132-mediated PINK1 deficiency induces pyroptosis of HCAECs

**Figure 5:**
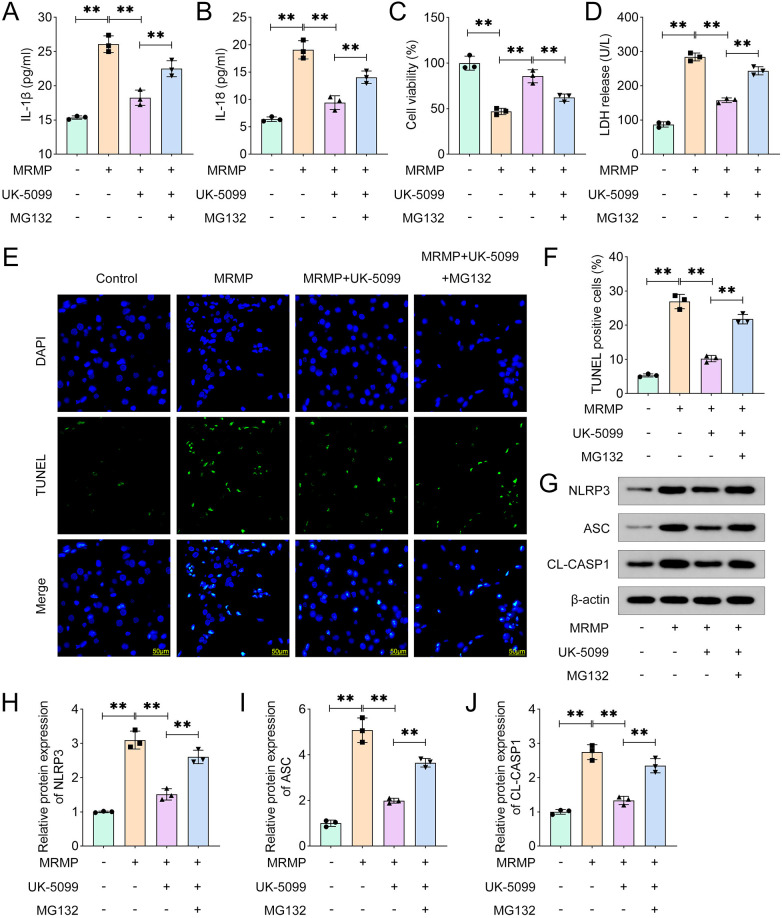
MG132-mediated PINK1 deficiency induces pyroptosis of HCAECs. **(A,B)** Cytokine release was determined using ELISA (*n* = 3). **(C)** Cell viability was determined using CCK-8 assay (*n* = 3). **(D)** Cytotoxicity was determined using LDH assay (*n* = 3). **(E,F)** Cell death was determined using TUNEL assay (*n* = 3). Scale bar: 50 μm. **(G–J)** Protein expression in MRMP-infected HCAECs was determined using Western blot (*n* = 3). **p < 0.01, one-way ANOVA followed by Tukey's *post hoc* test.

We found that MG132 significantly increased the release of IL-1β and IL-18 compared with MRMP plus UK-5099 group (Figures 5A,B). However, MG132 significantly suppressed the cell viability of HCAECs (Figure 5C). MG132 significantly increased the LDH levels in HCAECs (Figure 5D). Moreover, MG132 significantly increased the percentages of TUNEL positive cells (Figures 5E,F). Moreover, MG132 significantly alleviated the effects of UK-5099 and promoted the activation of NLRP3 inflammasome (Figures 5G–J). Therefore, inhibition of mitophagy contributes to the pyroptosis of HCAECs in MRMP.

### MPC1 mediates the pyroptosis of HCAECs via regulating PINK1-dependent mitophagy

**Figure 6:**
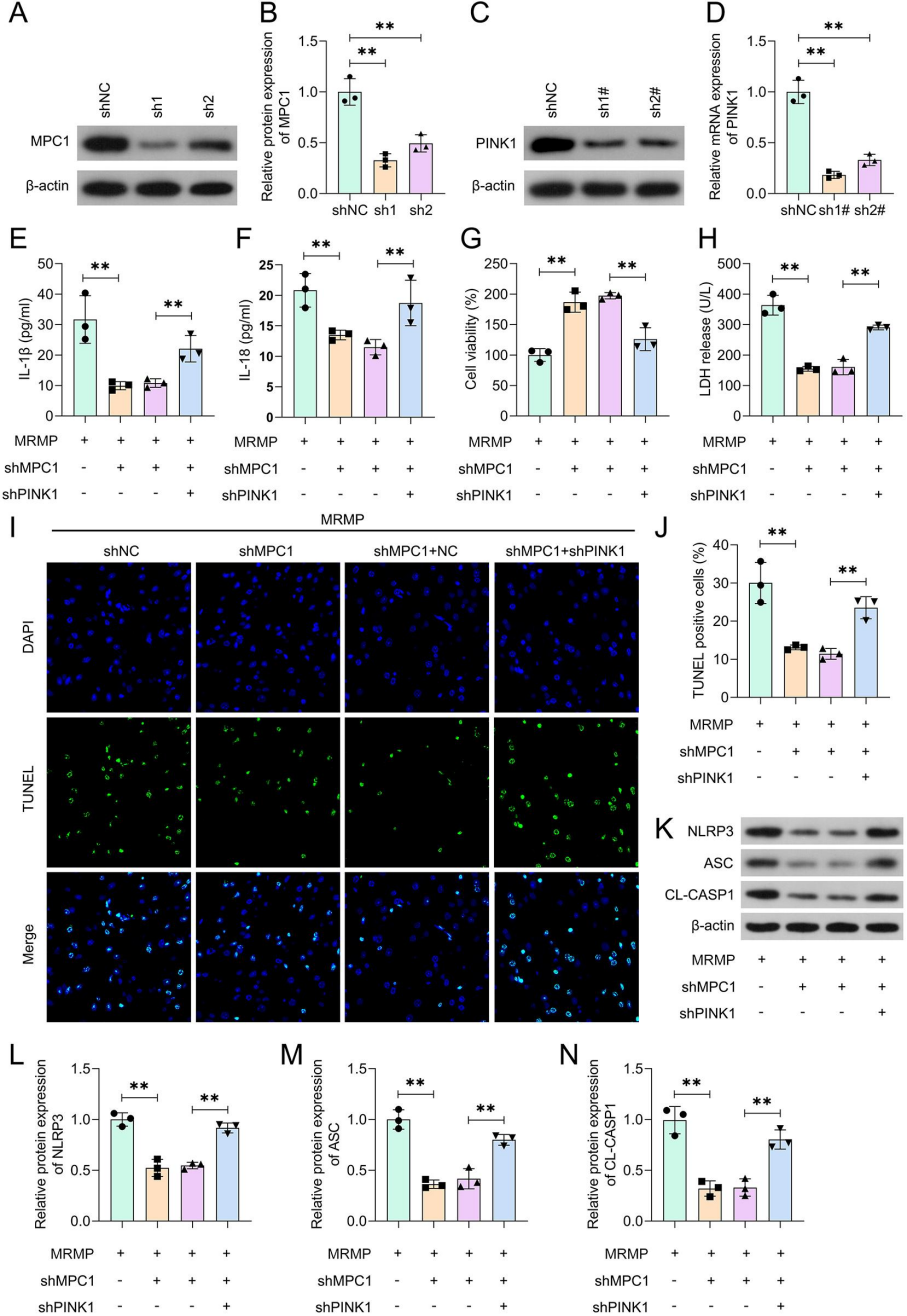
MPC1 induces pyroptosis of HCAECs via inhibiting PINK1-dependent mitophagy. **(A–D)** Protein expression in MRMP-infected HCAECs was determined using Western blot (*n* = 3). **(E,F)** Cytokine release was determined using ELISA (*n* = 3). **(G)** Cell viability was determined using CCK-8 assay (*n* = 3). **(H)** Cytotoxicity was determined using LDH assay (*n* = 3). **(I,J)** Cell death was determined using TUNEL assay (*n* = 3). Scale bar: 50 μm. **(K–N)** Protein expression in MRMP-infected HCAECs was determined using Western blot (*n* = 3). **p < 0.01, one-way ANOVA followed by Tukey's *post hoc* test.

To further confirm the role of MPC1/mitophag*y* axis in MRMP, HCAECs were transfected with the shRNA of MPC1/PINK1 respectively (Figures 6A–D). MPC1 knockdown significantly inhibited the release of IL-1β and IL-18 (Figures 6E,F), which was markedly reversed by shPINK1. shPINK1 significantly alleviated the effects of MPC1 knockdown and inhibited the cell viability of HCAECs (Figure 6G). shPINK1 significantly alleviated the effects of MPC1 knockdown and increased the release of LDH in HCAECs (Figure 6H). Moreover, shPINK1 significantly increased the death of HCAECs compared with shMPC1 + shNC group (Figures 6I,J). Additionally, MPC1 knockdown-mediated downregulation of NLRP3 inflammasome was significantly reversed by shPINK1. These results suggested that MPC1 mediates the pyroptosis of HCAECs via inhibiting mitophagy.

## Discussion

The work reported here establishes the mitochondrial pyruvate carrier-1 (MPC1) as a central metabolic–immunologic checkpoint that dictates the fate of HCAECs confronted with MRMP. Here, we reveal a previously unrecognised triad: (i) MRMP infection elicits rapid, resistance-specific transcriptional up-regulation of MPC1; (ii) MPC1 mediated inhibition of Parkin-dependent mitophagy, which attempts to constrain mitochondrial damage; (iii) mitophagy inhibition contributed to the activation of NLRP3 inflammasome and subsequent pyroptosis of HCAECs. Interestingly, pharmacological inhibition of MPC1 restores mitochondrial function and impeded the pyroptosis of HCAECs in MRMP.

MPC1 is dysregulated in various lung diseases, such as pulmonary hypertension, *Klebsiella pneumoniae*, and lung cancer (29–31). Here, we demonstrated that MPC1 was overexpressed in MRMP-infected HCAECs. However, why the host would up-regulate MPC1 when its primary function is to import pyruvate into mitochondria—an apparently pro-bacterial action. In this study, MRMP-mediated upregulation of MPC1 promoted mitochondria swelling and loss as well as inhibited the mitophagy in MRMP-infected HCAECs, contributing to cell pyroptosis.

Pyroptosis is characterized by activation of inflammasomes (32). NLRP3 inflammasome is abundantly expressed in vascular endothelial cells and *M. pneumoniae* (33, 34). NLRP3 cleaves inflammatory caspases, which drives N-terminal of gasdermin D (GSDMD-N) to cell membranes (35). The accumulation of GSDMD-N in membranes mediates pore formation, cell swelling and collapse, and subsequent pyroptosis (36). In this study, MPC1 deficiency alleviated MRMP induced activation of NLRP3 inflammasome and pyroptosis of HCAECs. Recently, increasing evidence has demonstrated that mitochondrial dysfunction mediates the enrichment of NLRP3 inflammasome is stimulated through “activating” (toll like receptor 4, STING) and “priming” signaling (adenosine triphosphate, oxidized mtDNA) (37, 38). Interestingly, maintaining mitochondrial homeostasis is a promising strategy for alleviating *M. pneumoniae*. Here, we demonstrated that MPC1 deficiency restored mitochondrial function and inhibited the activation of NLRP3 inflammasome. Therefore, MPC1 may promotes the progression of MRMP through regulating mitochondrial function.

Cellular mitophagy plays key roles in maintaining mitochondrial function (39). PINK1-mediated mitophagy regulates cellular homeostasis and alleviates programmed cell death, including pyroptosis (40, 41). Indeed, bacteria mediate fragmentation and ROS release in mitochondria, “priming” NLRP3 inflammation-mediated pyroptosis (42). Mitophagy signaling eliminates degraded mitochondria to remove ROS under a decrease of mitochondrial membrane potential (43). This metabolic boost mediated oxidative stress accumulation and *ΔΨ*m collapse, triggering the accumulation of mitochondrial PINK1 and cytosolic PARK2 (44). Interestingly, MPC1 deficiency mediated the activation of mitophagy, indicating that targeting MPC1 is necessary for maintaining mitochondrial function in MRMP-infected HCAECs.

Although pyroptosis is classically antimicrobial (45), *M. pneumoniae* is predominantly an extracellular pathogen. *M. pneumoniae* triggers excessive immune inflammatory response (46). Moreover, *M. pneumoniae* membrane lipoprotein components can interact with NLRP3 inflammasome (47), the activation of which is a key characteristic of pyroptosis (48). Here, we demonstrated that MRMP mediated mitochondria damage, impeded mitophagy, and activated NLRP3 inflammasome, resulting in the pyroptosis of HCAECs. A previous study has demonstrated that HCAEC pyroptpsis releases pro-inflammatory cytokines and exacerbates the progression of *M. pneumoniae*-induced Kawasaki disease (3). These findings suggested that pyroptosis may play a pro-inflammatory role in MRMP.

Several caveats merit discussion. First, our *in vitro* model lacks clinical practice and may underestimate the benefit of MPC1 inhibition. Second, although UK-5099 is the most widely used MPC inhibitor, off-target effects on mitochondrial complex I have been reported. Confirmation with next-generation compounds (e.g., MITO-66) is underway. Third, the secreted bacterial factors that initiate MPC1 transcription remain unidentified. Unbiased metabolomic profiling of MRMP conditioned medium is in progress. Finally, we did not investigate polymorphisms in the human MPC1 locus that could modulate susceptibility.

In summary, MRMP hijacks MPC1-driven metabolic reprogramming to trigger pyroptosis. MPC1 thus emerges as a tractable host-directed target whose manipulation could extend the lifespan of our current antimicrobial armamentarium against rising macrolide resistance.

## Data Availability

The raw data supporting the conclusions of this article will be made available by the authors, without undue reservation.
